# Case Report: Life-saving embolization: a rare case of post-traumatic retroperitoneal hematoma from deep circumflex iliac artery rupture

**DOI:** 10.3389/fsurg.2024.1468773

**Published:** 2024-12-13

**Authors:** Hong-wei Xu, Cong He

**Affiliations:** Department of Radiology, Shaoxing Second Hospital, Shaoxing, Zhejiang, China

**Keywords:** blunt abdominal trauma, retroperitoneal hematoma, deep circumflex iliac artery, transcatheter embolization, contrast-enhanced CT

## Abstract

**Introduction:**

Retroperitoneal hematoma with ongoing hemorrhage is a rare but critical condition following blunt abdominal trauma, requiring urgent evaluation and management. This case details a large retroperitoneal hematoma in the right iliac fossa caused by a rupture of the deep circumflex iliac artery (DCIA), successfully treated with transcatheter arterial embolization.

**Case description:**

A 66-year-old female presented to our hospital six hours after an electric tricycle accident with dizziness, fatigue, hypotension (80/50 mmHg), and tachycardia (105 beats/min). Laboratory tests revealed a hemoglobin level of 9.2 g/dl and a hematocrit level of 27.5%. Contrast-enhanced CT showed an 18 cm × 10 cm × 5 cm retroperitoneal hematoma in the right iliac fossa with active bleeding. Emergent angiography identified the bleeding source as a branch of the right DCIA. Embolization was performed using a microcoil through a coaxial microcatheter positioned proximal to the bleeding site, successfully stopping the hemorrhage. The patient's condition stabilized, and ultrasound monitoring showed a gradual reduction in hematoma size. The patient was discharged two weeks later.

**Conclusion:**

This case highlights a rare but severe instance of retroperitoneal hematoma due to DCIA rupture, effectively managed with transcatheter arterial embolization. The utility of contrast-enhanced CT and angiography in diagnosing active bleeding is emphasized, underscoring the efficacy of transcatheter embolization as a critical intervention in such life-threatening situations.

## Introduction

Retroperitoneal hematoma with ongoing hemorrhage represents a seldom encountered manifestation of acute abdominal pathology, posing a significant risk to patient survival necessitating expeditious and precise evaluation and management (1). This study presents a case of a sizable retroperitoneal hematoma situated in the right iliac fossa resulting from the rupture of a deep circumflex iliac artery (DCIA) following blunt abdominal trauma. The patient underwent emergent transcatheter endovascular embolization after computed tomography (CT) confirmed the presence of active bleeding within the hematoma, leading to successful treatment.

## Case description

**Figure 1:**
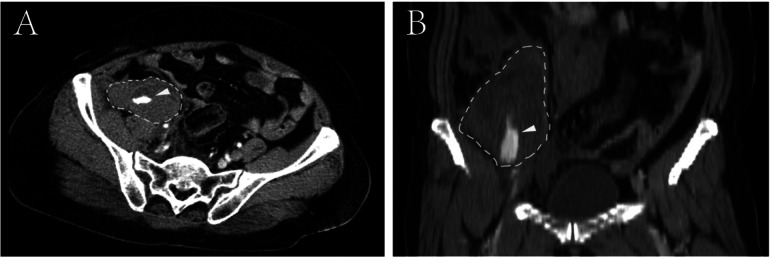
Contrast-enhanced computed tomography reveals a sizable retroperitoneal hematoma located in the right iliac fossa, exhibiting active contrast extravasation within the hematoma (arrowhead). **(A)** Cross-sectional CT image; **(B)** reconstructed coronal CT images.

**Figure 2:**
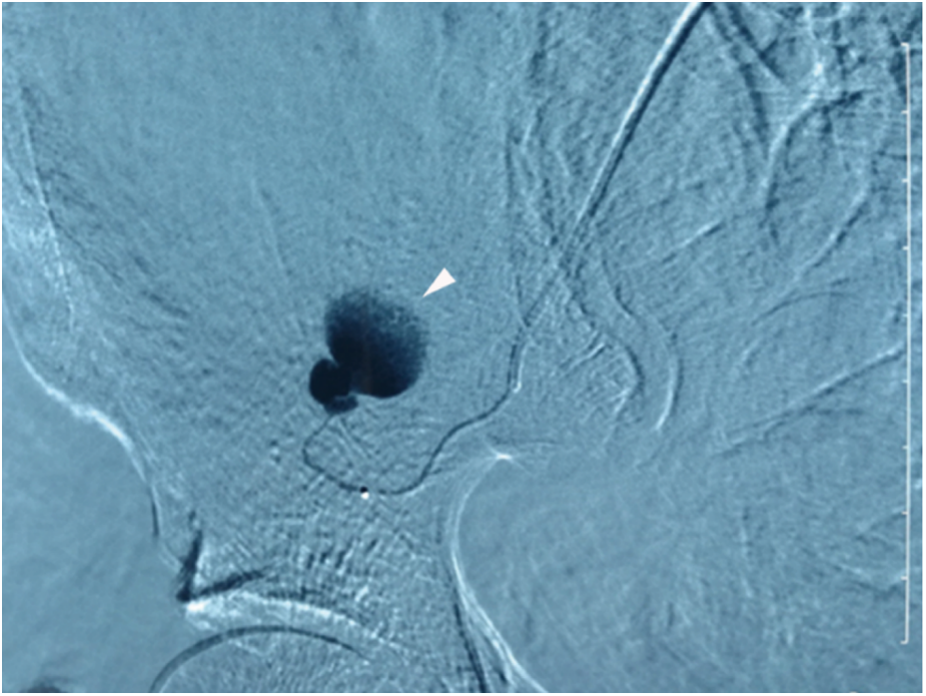
Superselective arteriography of the right DCIA confirms extravasation from a proximal branch, as indicated by the arrowhead. Additionally, observation of the disappearance of ascending branches of the DCIA suggests the presence of angiospasm.

A 66-year-old female patient was admitted to our hospital following a six-hour delay after an electric tricycle accident. The patient had no significant medical history. Also, there were no special personal or family illness histories. Upon admission, the patient presented with symptoms of dizziness and fatigue, along with a blood pressure of 80/50 mmHg and a pulse rate of 105 beats/min. Laboratory results indicated a hemoglobin level of 9.2 g/dl and a hematocrit level of 27.5%. The immediate contrast-enhanced CT imaging was performed, which revealed a sizable retroperitoneal hematoma measuring 18 cm × 10 cm × 5 cm in the right iliac fossa with evidence of contrast extravasation within the hematoma during the arterial phase. No apparent indications of rupture were observed in the liver, biliary system, pancreas, or spleen. However, the scan identified associated fractures, including a fracture of the 11th rib on the right side and transverse process fractures of the 1st, 2nd, and 4th lumbar vertebrae on the right side (Figure 1). Subsequent emergent angiography confirmed active bleeding from a branch of the right DCIA (Figure 2).

**Figure 3:**
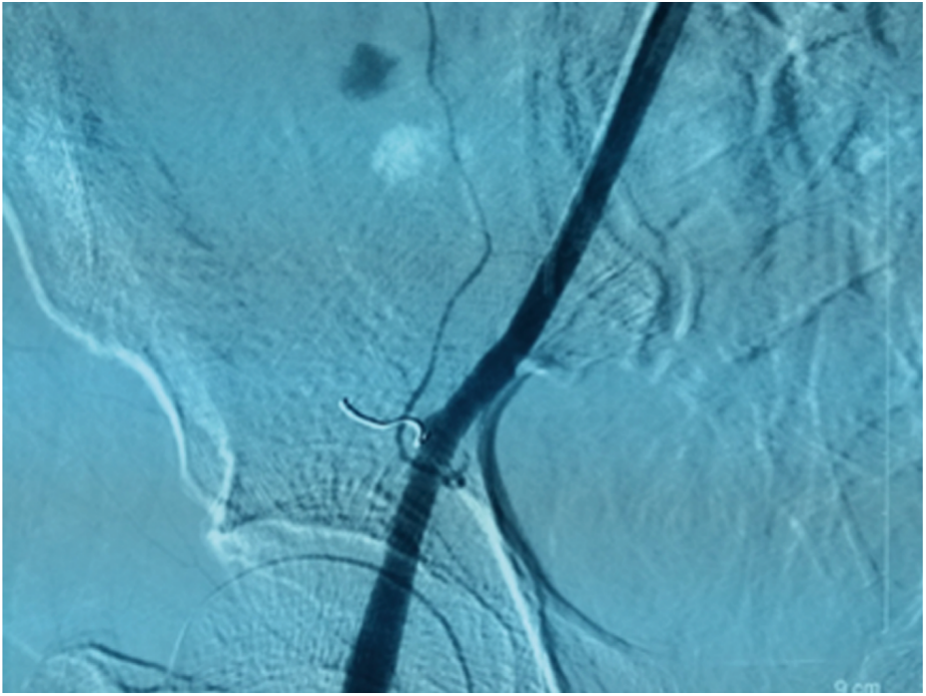
Following coil embolization, a right external iliac arteriogram revealed complete occlusion of the DCIA and resolution of the contrast extravasation.

Embolization was conducted utilizing a microcoil delivered through a coaxial microcatheter positioned proximal to the site of active hemorrhage (Figure 3). Specifically, the patient's blood pressure was stabilized using infusions and blood transfusions. Following the administration of local anesthesia, a percutaneous puncture of the left femoral artery was performed. A 5F arterial sheath was successfully inserted, through which a 4F Cobra catheter was introduced. Upon achieving superselective intubation, the active bleeding sites and responsible arteries were identified and embolized using a 2 mm × 20 mm microcoil. Treatment was concluded upon cessation of local contrast extravasation, resulting in prompt stabilization and gradual recovery of the patient. Ultrasound imaging was employed iteratively to assess the dimensions of the hematoma, which exhibited a progressive reduction. The patient was discharged from the medical facility two weeks subsequent to the procedure. To date, the patient remains asymptomatic.

## Discussion

Hematomas resulting from a rupture of the DCIA originating from the external iliac artery or femoral artery in the inguinal canal region are infrequent in clinical settings, encompassing spontaneous, iatrogenic, and traumatic occurrences (2). Spontaneous hemorrhage is the most frequently documented type, with numerous predisposing factors identified (3). Iatrogenic hemorrhage may arise following procedures such as laparotomy, paracentesis, and percutaneous drainage, primarily influenced by the selection of puncture site (4). Traumatic hemorrhage in DCIA is less common, with limited documentation in the literature (5).

The DCIA supply the internal oblique abdominis muscle and transversus abdominis muscle of the lateral abdominal wall, resulting in hematoma typically forming between these muscles following DCIA rupture (6). However, in the case presented, a traumatic retroperitoneal hematoma in the right iliac fossa exhibited active bleeding from a small proximal branch of the right DCIA as confirmed by angiographic examination. To the best of our knowledge, there have been no reports of retroperitoneal hematoma with active bleeding caused by rupture of the DCIA after blunt abdominal trauma.

CT and ultrasound are useful imaging modalities for diagnosing hematoma or hemorrhage. Contrast-enhanced CT can be used to evaluate active bleeding from ruptured arteries, and ultrasonography is especially useful for repeated noninvasive evaluations of hematomas (7).

Conservative treatment for this condition is acceptable if no hemodynamic dysfunction is present, or the hematoma is not increasing in size. However, under the conditions of hemodynamic instability, an expanding hematoma, or abnormal coagulation parameters, surgical or interventional treatment should be considered (4). Transcatheter arterial embolization has been described as an effective and less invasive method for controlling bleeding and avoiding surgical treatment in the management of active hemorrhage (7). Microcoils are frequently utilized in transcatheter arterial embolization, with reports suggesting that n-butyl 2-crylate (NBCA), gelatin sponge particles, and polyvinyl alcohol can also be effective materials for embolizing bleeding arteries (8). It is recommended that coils be placed at both the proximal and distal ends of the arterial bleeding site, although the selection of techniques and materials should be guided by angiographic assessments of vessel injury (9). In our particular instance, we encountered difficulty in advancing the microcatheter distally to the bleeding site of the minor branch of DCIA, so only proximal coiling was performed.

## Conclusion

In conclusion, we present a unique case of significant hemodynamic instability resulting from a ruptured and extensive hemorrhage of the distal branch of the inferior epigastric artery following blunt trauma to the abdomen. Subsequent emergency transcatheter endovascular embolization was successfully performed following the identification of active bleeding within the hematoma by computed tomography. Our findings highlight the utility of contrast-enhanced CT imaging and angiography in the accurate diagnosis of active bleeding, and underscore the efficacy of transcatheter embolization as a preferred therapeutic approach for managing the critical medical emergency.

## Data Availability

The original contributions presented in the study are included in the article/Supplementary Material, further inquiries can be directed to the corresponding author.
